# Bioconversion of Date Waste into Bacterial Nanocellulose by a New Isolate *Komagataeibacter* sp. IS22 and Its Use as Carrier Support for Probiotics Delivery

**DOI:** 10.3390/foods14162853

**Published:** 2025-08-18

**Authors:** Islam Sayah, Ibtissem Chakroun, Claudio Gervasi, Davide Barreca, Giovanni Lanteri, Daniela Iannazzo, Consuelo Celesti, Antonello Santini, Sami Achour, Teresa Gervasi

**Affiliations:** 1 Research Unit UR17ES30 “Genomics, Biotechnology and Antiviral Strategies”, Higher Institute of Biotechnology of Monastir, University of Monastir, Tahar Hadded Avenue, PB74, Monastir 5000, Tunisia; sayahislam2011@gmail.com (I.S.); samnaw2001@yahoo.fr (S.A.); 2 Department of Chemical, Biological, Pharmaceutical and Environmental Science, University of Messina, 98166 Messina, Italy; claudio.gervasi@unime.it (C.G.); davide.barreca@unime.it (D.B.); giovanni.lanteri@unime.it (G.L.); 3 Laboratory of Analysis, Treatment and Valorization of Environment Pollutants and Products, Faculty of Pharmacy, University of Monastir, Monastir 5000, Tunisia; chakrounibtissem2244@yahoo.fr; 4 Department of Engineering, University of Messina, Contrada Di Dio, 98166 Messina, Italy; daniela.iannazzo@unime.it (D.I.); consuelo.celesti@unime.it (C.C.); 5 Department of Pharmacy, University of Napoli Federico II, 80131 Napoli, Italy; 6 Department of Biomedical and Dental Sciences and Morpho functional Imaging, University of Messina, 98168 Messina, Italy

**Keywords:** bacterial nanocellulose, date waste, full factorial design, *Komagataeibacter* sp. IS22, cellulose-probiotic composite

## Abstract

Bacterial nanocellulose (BNC) has gained considerable interest over the last decade due to its unique properties and versatile applications. However, the low yield and the high production cost significantly limit its industrial scalability. The proposed study explores the isolation of new BNC producers from date palm sap and the use of date waste extract as a sustainable carbon source to improve BNC productivity. Results revealed three potential BNC producers identified as *Komagataeibacter* sp. IS20, *Komagataeibacter* sp. IS21, and *Komagataeibacter* sp. IS22 with production yield of 1.7 g/L, 0.8 g/L and 1.8 g/L, respectively, in Hestrin-Schramm (HS) medium. The biopolymer characterization indicated the presence of type I cellulose, a high thermal stability, and a highly dense network made of cellulose nanofibrils for all BNC samples. The isolate IS22, showing the highest productivity, was selected for an optimization procedure using a full factorial design with date waste extract as a carbon source. The BNC yield increased to 6.59 g/L using 4% date waste extract and 2% ethanol after 10 days of incubation compared to the standard media (1.8 g/L). Two probiotic strains, including *Bacillus subtilis* (BS), and *Lactobacillus plantarum* (LP) were successfully encapsulated into BNC matrix through a co-culture approach. The BNC-LP and BNC-BS composites showed antibacterial activity against *Pseudomonas aeruginosa*. BNC–probiotic composites have emerged as a promising strategy for the effective delivery of viable probiotics in a wide range of applications. Overall, this study supports the use of date waste extract as a sustainable carbon source to enhance BNC productivity and reduce the environmental footprint using a high-yielding producer (IS22). Furthermore, the produced BNC demonstrated promising potential as an efficient carrier matrix for probiotic delivery.

## 1. Introduction

Cellulose, the most abundant biopolymer existing in nature, is the main structural component of plant cell walls, providing strength and rigidity. It is widely used in the manufacture of several items like paper, hygienic products, and biomedical devices. Cellulose can also be produced from bacterial species belonging to the genera *Acetobacter*, *Gluconobacter*, *Komagataeibacter*, *Rhizobium*, *Agrobacterium*, and *Sarcina.* Fermented beverages and foods such as kombucha, vinegar, nata de coco—a translucent jelly food obtained from coconut water fermentation—and rotting fruits are considered the main habitat for these bacteria. In the presence of oxygen, they oxidize sugars and alcohols to acetic acid and generate, at the liquid air interface, an extracellular gelatinous layer made of crystalline cellulose to avoid desiccation and UV radiation [1].

Bacterial nanocellulose (BNC), a nanofibrous biopolymer composed of β-D-glucopyranose units linked by β-1,4 glycosidic bonds, exhibits enhanced characteristics compared to plant cellulose such as, non-allergenicity, high purity, a high degree of crystallinity (60–80%), mechanical strength (Young’s modulus of 15–30 GPa, resistance tensile strength), porosity and surface area that enables exceptional liquid retention, capable of holding up to 99% of its weight in liquids [2]. *Komagataeibacter xylinus* has been widely employed as a model strain for industrial-scale BNC production, due to its high productivity. Bacterial nanocellulose can be obtained in various shapes, each with distinct characteristics and applications. Under static conditions, the bacteria synthesize a gelatinous, rigid pellicle with a dense and highly organized nanofibril structure, whereas under shaking conditions, BNC is generated in the form of small beads with lower crystallinity and a weaker structure [3]. Hence, BNC has evolved as a highly demanded biomaterial with a wide range of applications.

Since 1992, the Food and Drug Administration has defined BNC as “generally recognized as safe” [4]. Therefore, BNC has been used in various food applications such as a thickening and stabilizing agent to improve the texture of fragile food hydrogels, like chocolate drink, ice cream, sausage, jam, etc. [5]. Additionally, BNC helps to maintain the sensorial properties of processed food over time by preserving the structural integrity [1]. Due to its high biocompatibility and water-holding capacity, BNC was further used in the cosmetics and skincare industry. The biopolymer showed a good performance over synthetic polymers in terms of skin adhesion and release control of bioactive molecules [6]. Functionalized BNC has been employed in bioremediation for adsorbing particular pollutants from the industrial waste stream [7]. In the biomedical area, particularly the field of wound treatment, BNC has evolved as a biomaterial of interest. Taking advantage of its biocompatibility and porosity, BNC showed a high ability to absorb wound exudate, stimulate the re-epithelization by maintaining the gaseous exchange and the immobilization of antimicrobial agents [8].

Despite these advances, the large-scale production of BNC remains limited by high fermentation costs and low product yield. Therefore, to address these issues, several studies have focused on the isolation of new bacterial strains with high production capabilities from various sources, such as fermented beverages, vinegar, flowers, and fruits [9,10,11,12]. While other attempts have been oriented to the use of sustainable materials derived from agro-industrial wastes and industrial by-products as alternative substrates for BNC synthesis, such as fruit peels and juice [13,14], by-products of the dairy industry [15], soybean oil refinery effluent [16], and textile effluent [17].

The present study aims to isolate highly productive bacterial strains from Tunisian date palm sap and evaluate the use of date waste extract as a sustainable and economical carbon source to enhance BNC production. Date palm sap, locally known as *lagmi* in Tunisia, is a sweet beverage (containing from 92 to 95% sugars) exuded from the trunk of the date palm tree, rich in yeasts, lactic acid bacteria and acetic acid bacteria, which represent a promising source for potential BNC-producing candidates [18]. The isolated strains were identified through morphological, biochemical, and molecular characterization. The resulting BNC pellicles were characterized with Fourier Transform Infrared spectra (FTIR), Scanning Electron Microscopy (SEM), and Thermogravimetric Analysis (TGA). This work evaluates the potential use of date waste extract, which should be disposed of as a sustainable and economic carbon source to enhance BNC production. In Tunisia, the date industry releases around 25,000 tons of date by-products annually throughout the various stages of processing, posing a significant environmental risk. The richness of date fruits, especially in carbohydrates (44–88%), dietary fiber (6.4–11.5%), and minerals (such as calcium, magnesium, potassium, and iron), as well as vitamins (B1, B2), fatty acids (0.2–0.5%), protein (2.3–5.6%) and polyphenols, make date extract a good alternative media for BNC biosynthesis [19]. The BNC production process was optimized using a full factorial design (FFD), and the resulting material was further evaluated as a probiotic carrier by immobilizing *Bacillus subtilis* and *Lactobacillus plantarum* through a co-culture approach. The viability of the immobilized probiotic cells was assessed by cell counting on MRS agar. The antibacterial activities of the functionalized BNC biofilms were tested against *Escherichia coli*, *Staphylococcus aureus*, and *Pseudomonas aeruginosa* using the agar diffusion assay.

## 2. Materials and Methods

### 2.1. Sample Collection and Microbial Strains

A palm sap sample was purchased from a local market in Nefta, Southern Tunisia. Upon collection, the sample was stored in sterile glass bottles at 4 °C and used for fermentation within 24 h at room temperature.

Two different varieties of low-grade date fruit (Alig, Kentichi) were obtained from a local market in Nefta, Southern Tunisia. The fruits were washed, pitted, and stored at 4 °C in sterile polyethylene bags until use, within 48 h. These varieties were selected due to their classification as low-grade fruits, typically regarded as by-products of the Tunisian date industry, where they are predominantly utilized for animal feed or for valorization into value-added products [19].

*Bacillus subtilis* (BS) and *Lactobacillus plantarum* (LP) were obtained from commercially available suspensions. The pathogenic bacteria used for susceptibility studies were *E. coli* ATCC 25922, *S. aureus* ATCC 25923, and *P. aeruginosa* ATCC 27853.

### 2.2. Culture Media

The screening media used for the selection of cellulose-producing strains were standard Hestrin-Schramm (HS) medium and GEY (Glucose, Ethanol and Yeast extract) agar. HS medium consists of 2% (*w*/*v*) glucose, 0.5% (*w*/*v*) peptone, 0.5% (*w*/*v*) yeast extract, 0.27% (*w*/*v*) disodium phosphate and 0.115% (*w*/*v*) citric acid monohydrate [20]. The initial pH of the medium was adjusted to 6 with 1 mol/1 acetic acid. For the solid HS medium, 1.5% (*w*/*v*) agar was added. GEY agar plates contain 2.0% D-glucose, 1.0% yeast extract, 5% ethanol, 0.3% CaCO_3_ and 2% agar. Sodium hydroxide solution (1 M) was employed for bacterial nanocellulose purification.

For the cultivation of probiotic strains, de Man, Rogosa and Sharpe (MRS; Oxoid, Milan, Italy) broth and agar were used. These microorganisms were incubated in MRS broth at pH 7.0 and 37 °C under anaerobic conditions for 18–20 h.

For susceptibility assays, Mueller–Hinton Broth (MHB; Oxoid, Milan, Italy) and Mueller–Hinton Agar (MHA; Sigma, Milan, Italy) were employed. Pathogenic bacteria were grown in MHB at 37 °C for 18–20 h prior to testing.

### 2.3. Screening and Isolation of Cellulose-Producing Bacteria

A gel pellicle sample of naturally fermented palm sap was transferred under sterile conditions into a flask, containing sterile saline and glass beads and subsequently shaken at 30 °C for 24 h. The resulting cell suspension was serially diluted and spread on GEY agar and incubated at 30 °C for 2–5 days. Colonies that produced a clear zone of CaCO_3_ solubilization on the agar medium were selected and purified by repeated streaking on the HS agar plates. The selected strains were seeded in test tubes containing 5 mL of HS medium and incubated statically at 30 °C for 15 days. Only the bacterial isolates that showed the pellicle formation at the air–liquid interface were considered as potential cellulose-producing bacteria [21]. The resulting pellicles were manually collected and washed with distilled water to eliminate any residual medium components. The pellicles were then boiled in 1% (*w*/*v*) NaOH solution at 80 °C for 1h to eliminate the attached bacterial cells. After several washings with distilled water (2–5 times), the purified films were vacuum-dried and weighed to determine BNC production (g/L). Finally, the bacterial isolates exhibiting the highest BNC yield were identified as the most promising cellulose producers.

### 2.4. Identification of Cellulose-Producing Bacteria

The cellulose-producing isolates were Gram-stained, and cell morphology was examined under a light stereomicroscope (Nikon SMZ1500, Tokyo, Japan). The biochemical characteristics were assessed using API system test strips (BioMerieux, Lyon, France). The API 20 E kit was used according to the method described by Boone [22]. Genomic DNA was extracted using GeneJET Genomic DNA Purification Kit (Thermo Scientific™, Waltham, MA, USA) following the manufacturer’s instructions for Gram-negative bacteria. The 16S rRNA gene sequence was amplified using universal primers 27F (5′-AGAGTTTGATCMTGGCTCAG-3′) and 1492R (5′-TACGGYTACCTTGTTACGACTT-3′) [23], producing an amplicon of approximately 1500 bp. PCR was conducted using DreamTaq Green PCR Master Mix (Thermo Scientific™, Waltham, MA, USA) in a final volume of 50 µL. The PCR cycling parameters consisted of an initial denaturation at 95 °C for 5 min, followed by 35 cycles of denaturation at 95 °C for 45 s, annealing at 55 °C for 45 s, elongation at 72 °C for 60 s, and a final extension step at 72 °C for 5 min. PCR products were assessed on a 1.5% (*w*/*v*) agarose gel, and the concentration and purity were verified using an N50 NanoPhotometer (Implen, Westlake Village, CA, USA). Purified DNA fragments were sequenced bidirectionally by Macrogene Europe (Milan, Italy) using the same forward and reverse primers used for PCR amplification.

Sequence alignments were performed with the ClustalW algorithm (https://www.genome.jp/tools-bin/clustalw, accessed on 4 December 2024), and sequence similarity was determined using the Basic Local Alignment Search Tool (BLAST, http://blast.ncbi.nlm.nih.gov/, accessed on 4 December 2024) against the National Center for Biotechnology Information (NCBI; https://blast.ncbi.nlm.nih.gov/Blast.cgi, accessed on 4 December 2024) database to calculate statistical significance of the matches. Evolutionary analyses were conducted in MEGA 11 using the Neighbor-Joining method and the Tamura model [24] with a bootstrap of 1000 replicates. The resulting sequences were compared to type strains of the *Komagataiebacter* genus collected from the NCBI database.

### 2.5. Analysis of BNC Pellicles

#### 2.5.1. Structural Analysis: Fourier Transform Infrared Spectroscopy

The chemical composition of the dry BNC pellicles produced by the strains IS20, IS21, and IS22 was examined by FTIR. Infrared spectra were recorded using a Fourier-Transform Infrared Spectrum Two Spectrometer (PerkinElmer Inc., Waltham, MA, USA), coupled with the attenuated total reflectance (ATR) method within the range of 4000 to 500 cm^−1^. The obtained spectra were constructed using Origin Pro 9.0 software. To study the crystallinity properties, total crystallinity index (TCI), lateral order index (LOI) and hydrogen bond intensity (HBI) [25] were calculated from the absorbance ratios A_1372_/A_2900,_ A_1429_/A_897_, and A_3400_/A_1320_, respectively.

#### 2.5.2. Thermogravimetric Analysis

The thermal stability of BNC pellicles was investigated by a Perkin Elmer TGA 4000 instrument (PerkinElmer Inc., Waltham, MA, USA). The samples were weighed and placed on an aluminum tray with a weight range of 5–10 mg. Then, they were heated under argon flow (20 mL/min) from 100 °C to 400 °C, at a heating rate of 5 °C/min. Each sample was analyzed in triplicate.

#### 2.5.3. Scanning Electron Microscopy

The Surface morphology of BNC pellicles was studied using scanning electron microscopy EVO MA10 SEM (Zeiss, Jena, Germany). Before imaging, the samples were dehydrated via lyophilization and coated with a sputtered palladium-gold layer (20 ± 5 nm). Imaging was performed at an accelerating voltage of 20 kV with different magnifications of 1 µm, 2 µm and 10 µm.

### 2.6. Evaluation of Different Date Extracts for BNC Production

Two different varieties of low-grade date fruit (Alig, Kentichi) were evaluated for their potential as an alternative medium for BNC production by the isolate *Komagataeibacter* sp. IS22. Both extracts were prepared using an ultrasound-assisted extraction at a temperature of 40 °C for 20 min, maintaining a liquid-to-solid ratio of 10 mL/g. This procedure was carried out following the methodology described by Sayah et al. [19].

The BNC production was carried out using date extracts as both a carbon source and the sole nutrient source in comparison to the standard media (HS) under static conditions (10 days, 30 °C). The best alternative medium with the highest productivity was selected for further optimization procedure using FFD.

### 2.7. Optimization of BNC Production Using Full Factorial Design

The biosynthesis of BNC is influenced by multiple operational parameters such as the bacterial strain, the culture conditions, and the medium composition. In this work, the effects of incubation time, date waste extract, and ethanol on BNC productivity were investigated to determine the optimum conditions for a maximum yield. The isolate IS22, which yielded the highest BNC production, was selected for optimization using date waste extract as a sustainable carbon source under static culture conditions. A FFD with three factors and two levels (−1, +1) was used for this purpose. Table 1 presents the experimental matrix with coded and uncoded levels, including the incubation time (X_1_, ranging from 5 to 10 days), date extract (X_2_, ranging from 2% to 4%), and the ethanol percentage (X_3_, ranging from 1 to 2%). All experiments were conducted in triplicate, and the dry BNC yield (g/L) was considered the response value. Incubation temperature, pH and inoculum size were maintained constant as follows: 30 °C, 6 and 10%. All statistical analyses were carried out via Minitab 21 statistical software. A statistical model was developed to evaluate the correlation between the independent variables and the corresponding response. The significance of the model was estimated based on the coefficient of determination (R^2^) and the *p*-value. Analysis of variance was used to evaluate the significance of the independent variables. Finally, to validate the suggested model, the experiment was performed under the optimum conditions and compared to the predicted value given by the model.

### 2.8. Immobilization of Probiotic Strains into BNC Matrix

#### 2.8.1. Co-Culture

Immobilization of probiotic cells within the BNC matrix was performed as described by Sabio et al. [26]. Briefly, 0.1 mL of IS22 suspension (OD_600_ = 0.3) was co-cultured with 0.1 mL of a probiotic suspension (OD_600_ = 0.4) in 1 mL of HS medium under aerobic conditions at 30 °C for 3 days, during which a BNC pellicle was formed at the air-liquid interface. Subsequently, the HS medium was replaced with 5 mL of MRS broth and incubated in anaerobic conditions at 37 °C for 2 days, with the MRS broth being refreshed after the first day. After this incubation, the functionalized BNCs were obtained. Control samples were prepared following the same procedure, without probiotics addition.

#### 2.8.2. Enumeration of Probiotic Cells

The concentration of probiotic cells embedded within the BNC matrix was determined by serial dilution and plating on MRS agar, followed by incubation at 37 °C for 48 h.

Bacterial counts were expressed as mean log colony-forming units (log CFU) per mL of sample.

#### 2.8.3. Evaluation of Antibacterial Activity of BNC-Probiotic Composites

The antimicrobial activity of functionalized BNC was assessed using the agar diffusion method. Briefly, 0.1 mL of an overnight culture of *E. coli* ATCC 25922, *S. aureus* ATCC 25923, and *P. aeruginosa* ATCC 27853 was uniformly spread on MHA plates. Then, BNC-BS and BNC-LP were placed on the inoculated plates. Free probiotics BNC and free probiotic suspension were also tested under the same conditions. Following incubation for 24 h at 37 °C, the diameters of the inhibition zones were measured and compared.

### 2.9. Statistical Analysis

Statistical analysis was performed using Minitab software (version 19.1). One-way ANOVA was conducted at a 95% confidence level (*p* < 0.05).

All experiments were carried out in triplicate, and results are presented as mean ± standard deviation (SD). Prior to ANOVA, data were evaluated for normality and the presence of outliers using the built-in diagnostic tools provided by Minitab. No significant deviations from normality or extreme outliers were observed, and therefore, no data transformation was applied.

## 3. Results and Discussion

### 3.1. Isolation and Identification of Cellulose-Producing Bacteria

**Figure 1:**
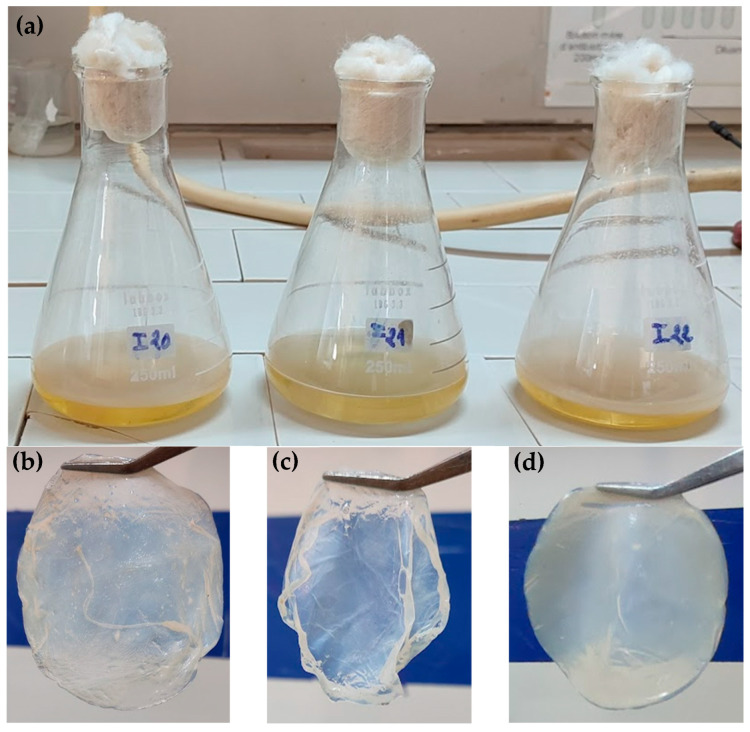
Bacterial nanocellulose production in HS medium under static culture (**a**), BNC pellicles synthesized by the strain IS20 (**b**), IS21 (**c**), and IS22 (**d**).

A total of twenty-two bacterial isolates were obtained from date palm sap. All of them showed clear zones around the developed colonies, indicating their ability to produce acetic acid, which dissolves CaCO_3_ in the medium. They were then tested for their ability to produce bacterial nanocellulose by inoculating them in HS broth under static conditions at 30 °C for 15 days. Among them, only three isolates named IS20, IS21 and IS22 produced a pellicle at the air-liquid interface, as shown in Figure 1. After five days of incubation on HS agar, these isolates displayed creamy-colored, smooth, convex, translucent colonies with a circular shape and entire margins. Dispersion of the colonies using an inoculation loop was challenging because of their hard texture, likely caused by the cellulose matrix they produced. All the bacterial isolates were Gram-negative, rod-shaped, non-motile and occurred singly or in pairs (Table 2). Furthermore, the selected strains were evaluated for their enzymatic and biochemical profile. Briefly, all strains revealed negative response to indole, urease test, citrate utilization, gelatin liquefaction, ornithine decarboxylase, nitrate reduction and hydrogen sulfide production. Strains IS22 and IS21 showed a positive response to Voges-Proskauer, whereas IS20 showed a negative one. All strains were able to produce acid from glucose, melibiose and arabinose. Strains IS22 and IS20 were not able to produce acid from rhamnose, while IS21 oxidizes rhamnose into acid. Additionally, all strains showed positive results for catalase activity, overoxidation of ethanol, but negative for the oxidase test. To confirm these results, the 16S rRNA gene sequencing was performed, and sequences were deposited in the GenBank nucleotide database under the accession numbers PQ678922, PQ678955, and PQ678840 for strains IS20, IS21, and IS22, respectively.

**Figure 2:**
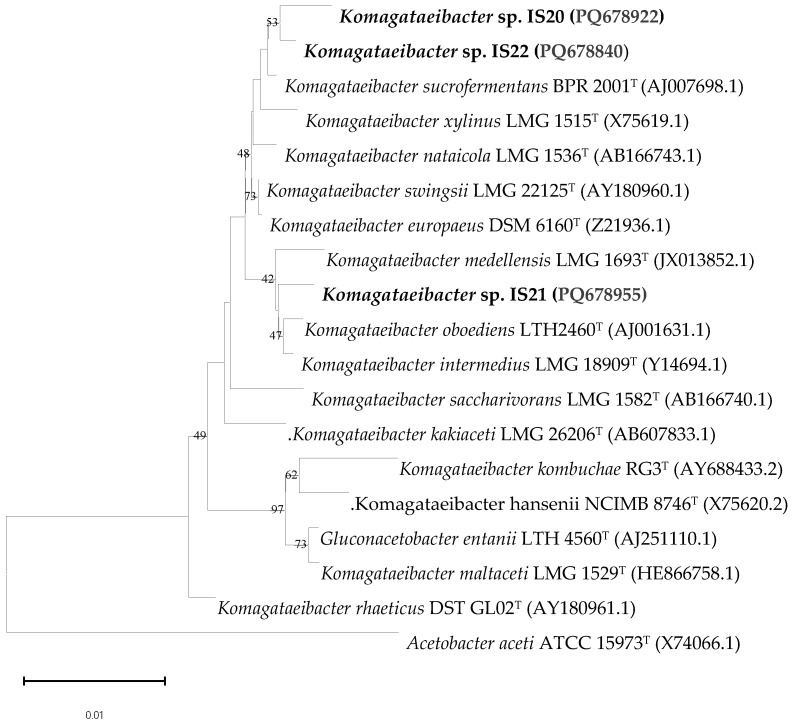
Phylogenetic tree based on 1275, 1278, and 1352 unambiguous nucleotides of the 16S rRNA gene sequences, showing the position of strain IS20, IS21 and IS22 among related species of the genus *Komagataeibacter*. GenBank accession numbers are given in parentheses. The Neighbor joining method was used for tree reconstruction. *Acetobacter aceti* was used as an outgroup. Bootstrap values are indicated at branching points. Bar, one substitution per 100 nucleotides.

The phylogenetic analysis indicated that strains IS20, IS21 and IS22 are closely related to the genus *Komagataeibacter*, with sequence similarities of 99.76%, 99.53% and 99.48% respectively (Table 2). Based on the phylogenetic reconstruction (Figure 2), isolates IS20 and IS22 revealed the highest similarity with the species *Komagataeibacter sucrofermentans* BPR 2001^T^, while isolate IS21 showed greater similarity to the species *Komagataeibacter intermedius* LMG 18909^T^. In addition, some variations in the BNC yield were observed among the isolated strains. Isolates IS22 and IS20 possess the highest BNC productivity with yields of 1.8 g/L and 1.7 g/L, respectively, while isolate IS21 recorded the lowest BNC production at 0.8 g/L. The difference between IS20 and IS22 was not significant (*p*-value = 0.14), while the yield of isolate IS21 was significantly lower than that of both IS20 and IS22, with a *p*-value < 0.001. These findings show the high variability of species belonging to the genus *Komagataeibacter* in terms of cellulose productivity.

In the same context, many researchers have reported the isolation of different species of BNC producers with different synthesis yields. Suwanposri et al. reported the identification of cellulose-producing bacteria from tropical fruits like papaya, watermelon, mangosteen and rambutan, producing 1.15 g/L [12]. Park et al. isolated *Gluconacetobacter hansenii* from rotten apples, achieving a production yield of 0.35 g/L of BNC [27]. In addition, *Gluconacetobacter xylinus* was isolated from kombucha, producing 0.28 g/L of BNC after 7 days of cultivation at 30 °C under static conditions [9]. Rangaswamy et al. reported a BNC yield of 4.7 g/L using *Gluconacetobacter* sp. RV28 isolated from a rotten pomegranate in an optimized medium [28]. Similarly, Yang et al. indicated that *Gluconacetobacter xylinus* CH001 produced 2.53 g/L of BNC from litchi extract [29].

The isolation of BNC-producing bacteria from Tunisian date palm sap, with high production yields, is reported here for the first time.

Following the isolation and identification of BNC-producing isolates, structural and thermal analyses were performed on the cellulose pellicles to evaluate their suitability for industrial and biomedical applications.

**Table 2:** Differential phenotypic characteristics of isolates IS22, IS20, IS21 and other related type strains of *Komagataeibacter* species.

	*Komagataeibacter* sp. IS22	*Komagataeibacter* sp. IS20	*Komagataeibacter sucrofermentans* BPR 2001^T^ (AJ007698.1) [30]	*Komagataeibacter xylinus* LMG 1515^T^ (X75619.1) [31]	*Komagataeibacter* sp. IS21	*Komagataeibacter oboediens* LTH2460^T^ (AJ001631.1) [32]	*Komagataeibacter medellensis* LMG 1693^T^ (JX013852.1) [33]	*Komagataeibacter intermedius* LMG 18909^T^ (Y14694.1) [34]
16S rRNA similarity with IS22 (%)	100	99.30	99.48	99.26	98.68	99.04	98.74	99.04
16S rRNA similarity with IS20 (%)	99.30	100	99.76	99.45	99.21	99.21	98.98	99.21
16S rRNA similarity with IS21 (%)	98.68	99.21	99.14	98.98	100	99.53	99.30	99.53
Morphology	rod shaped	rod shaped	rod shaped	rod shaped	rod shaped	rod shaped	rod shaped	rod shaped
Gram staining	−	−	−	−	−	−	−	−
Catalase	+	+	+	+	+	+	+	+
Oxidase	−	−	−	−	−	−	−	−
Brown pigment production	−	−	−	−	−	−	−	−
Indole	−	−	NR	NR	−	NR	NR	NR
Voges Proskauer	+	−	NR	NR	+	NR	NR	NR
Urease test	−	−	NR	NR	−	NR	NR	NR
Citrate utilization	−	−	NR	NR	−	NR	NR	NR
Gelatin liquefaction	−	−	NR	NR	−	NR	NR	NR
Hydrogen sulfide production	−	−	NR	NR	−	NR	NR	NR
Ornithine decarboxylase	−	−	NR	NR	−	NR	NR	NR
Nitrate reduction	−	−	NR	NR	−	NR	NR	NR
Overoxidation of Ethanol	+	+	+	NR	+	+	+	+
Motility	−	−	−	−	−	−	−	−
BNC Production yield (g/L)	1.8	1.7	1.72 [35]	1.83 [36]	0,8	No	No	5.93 [37]
Acid production from								
Glucose	+	+	+	+	+	+	+	NR
Sucrose	−	−	+	+	−	+	+	NR
Glycerol	+	+	+	+	+	+	NR	NR
Mannitol	−	−	NR	NR	−	−	+	NR
Mannose	−	−	NR	NR	−	NR	NR	NR
Inositol	−	−	NR	NR	−	NR	NR	NR
Sorbitol	−	−	NR	NR	−	−	+	NR
Rhamnose	−	−	NR	NR	**+**	NR	NR	NR
Melibiose	+	+	NR	NR	+	NR	NR	NR
Amygdaline	−	−	NR	NR	−	NR	NR	NR
Arabinose	+	+	NR	NR	+	NR	NR	NR

NR = Not Reported.

### 3.2. Characterization of BNC Pellicles

#### 3.2.1. Fourier Transform Infrared Spectroscopy

**Figure 3:**
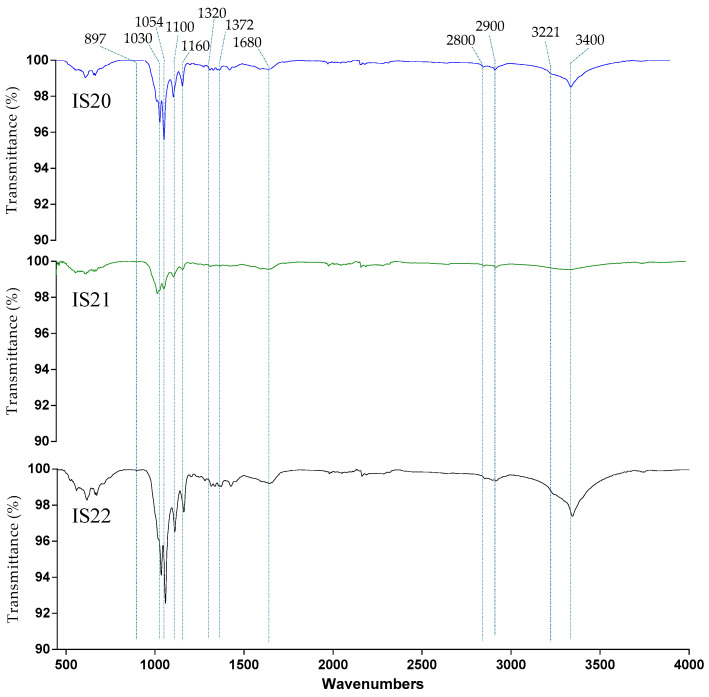
FTIR spectra of BNC pellicles synthesized by the strains IS20, IS21 and IS22.

FTIR spectroscopy was used to evaluate the structural differences of BNC pellicles obtained from strains IS20, IS21, and IS22. As shown in Figure 3, the FTIR spectra of all samples displayed the characteristic bands of cellulose, represented by the O–H stretching at 3400 cm^−1^ due to the free hydroxyl groups of the natural polysaccharide and the inter- and intra-molecular hydrogen bond vibrations of cellulose [38]. The samples also exhibited additional bands, like those attributed to the C-H stretching vibration of -CH_2_ and -CH_3_ of the sugar rings at around 2800 cm^−1^ [39], and a band around 1680 cm^−1^ related to H-O-H bending of absorbed water [40]. The fingerprint region displayed four strong bands corresponding respectively to the C–O–C asymmetric stretching of the β-glycosidic bond at 1160 cm^−1^ [41], C–C bonds of the monomer units of polysaccharide at 1110 cm^−1^ [42], C–O–C pyranose ring stretching vibration around 1054 cm^−1^ [43], and C–O symmetric stretching of the primary alcohol at 1030 cm^−1^ [44].

Furthermore, the FTIR spectra could provide valuable insight into the crystallinity index of the sample. The band at around 1420–1430 cm^−1^ reflects the amount of the crystalline structure in the BNC samples, while the band at 897 cm^−1^ is assigned to the amorphous region [45]. Nelson and O’Connor have also proposed that the ratio between the latter two bands can be utilized as an empirical crystallinity index and a lateral order index [25]. Moreover, the ratio between the bands at 1372 and 2900 cm^−1^ provides an estimate of the TCI. The HBI of cellulose, calculated as the ratio between the absorbance bands at 3400 and 1320 cm^−1^, provides information on the distribution of cellulose fibrils. In fact, the high interaction between the adjacent chains, resulting in a strong hydrogen bond, demonstrates high mechanical and thermal properties [46,47]. According to the data reported in Table 3, all samples demonstrated a similar crystallinity index with no significant difference between the values of LOI, HBI and TCI (*p*-value = 0.21 > 0.05), which may be due to the close relationship between the producer strains. In addition, the presence of the two crystalline cellulose allomorphs, cellulose Iα and cellulose Iβ, can be investigated by examining two characteristic bands that occur in the region between 3220 and 3280 cm^−1^ [48]. A very small peak, typically shifted to lower wavenumbers at around 3221 cm^−1^, is attributed to hydrogen bonds present only in cellulose Iα [48], whereas the band closer to 3277 cm^−1^ is proportional to the amount of monoclinic cellulose Iβ. Based on the obtained results, all samples showed a very small band at a wavelength around 3221 cm^−1^ due to intramolecular hydrogen bonds characteristic of triclinic Iα cellulose.

#### 3.2.2. Thermal Properties: Thermogravimetric Analysis

**Figure 4:**
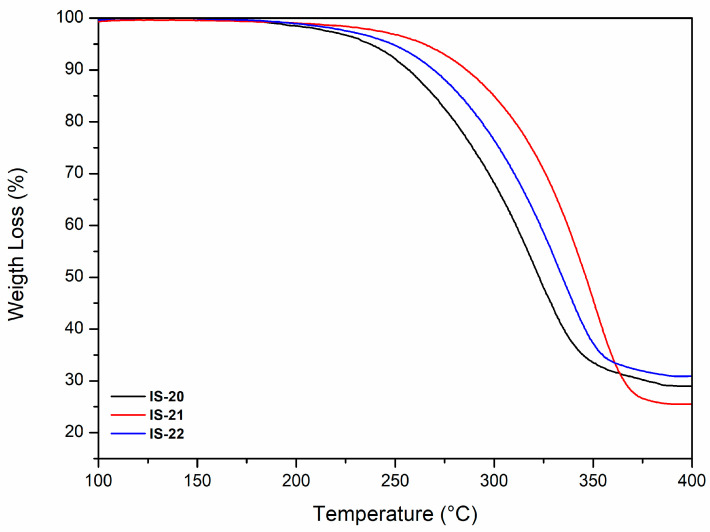
TGA curves of BNC pellicles produced by the strains IS20, IS21 and IS22.

The thermal stability of BNC pellicles produced by strains IS20, IS21, and IS22 was evaluated using thermogravimetric analysis. The TGA thermograms (Figure 4) revealed a marked weight reduction occurring from 250 °C to 400 °C, primarily due to the decomposition and degradation of cellulose, particularly the breakdown of small functional groups such as hydroxyl and methyl hydroxyl moieties [49,50]. This process resulted in a weight loss of approximately 70%, 75%, and 68% for IS20, IS21 and IS22 samples, respectively. The differences observed among the analyzed samples may be attributed to several factors, including the bacterial strain, molecular weight, BNC fibril distribution, and the crystallinity index of BNC [51]. Cheng et al. previously reported that BNC material undergoes a two-step thermal degradation process: the first stage involves the decomposition of the short fragments (200 °C to 360 °C), followed by the breakdown of the longer polymeric chains and the six-membered pyran ring cyclic structures (360 °C to 600 °C) [50]. In the present study, a single degradation stage was detected, likely attributable to the limited scanning temperature range.

#### 3.2.3. Microstructure: Scanning Electron Microscopy

**Figure 5:**
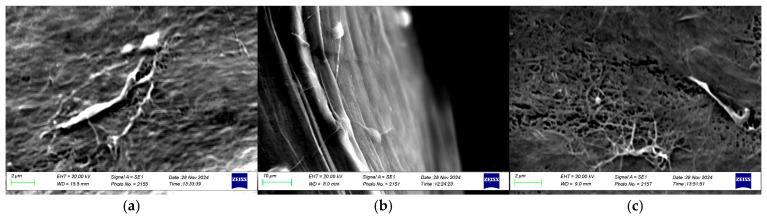
SEM image of BNC pellicles produced by strains IS20, IS21, and IS22. BNC network (**a**,**c**), (**b**) cellulose nanofibrils.

The morphological microstructure of BNC films synthesized under static conditions by strains IS20, IS21, and IS22 was examined using Scanning Electron Microscopy and is presented in Figure 5. All samples showed a highly dense fibrous network composed of interwoven cellulose microfibrils, tightly connected to form a consistent structure. SEM images revealed a similar distribution of BNC fibrils among the selected strains, which may be attributed to the high sequence similarity between them. As shown in Figure 5b, the cellulose microfibrils were secreted in the extracellular compartment and interconnected by hydrogen bonds to create a three-dimensional network (Figure 5a,c). These findings correspond to those reported in the literature [52].

Given the promising properties of the BNC produced by strains IS20, IS21, and IS22, the following experiments were conducted to enhance production efficiency using low-grade date fruit extracts.

### 3.3. Screening of Date Waste Extracts for BNC Production

In this work, date extracts prepared from two low-grade date fruit varieties were used as a sustainable carbon source for BNC production. As shown in Table 4, the highest BNC yield was obtained using HS media supplemented with Alig extract (HS + AE) as a carbon source, followed by the HS supplemented with Kentichi extract (HS + KE). The standard medium showed the lowest BNC productivity of 1.8 g/L. These results may be due to the richness of both extracts in simple sugar, minerals, and polyphenols, which improve the biomass growth and therefore the BNC yield. Conversely, when date extracts were used as the sole source of nutrients (AE, KE) without any additives, a lower BNC yield was observed compared to the standard (HS). This reduction may be attributed to the lack of a nitrogen source, essential for bacterial growth. Thus, HS supplemented with Alig extract was considered for further optimization via FFD.

### 3.4. Optimization of BNC Production Using Full Factorial Design (FFD)

**Figure 6:**
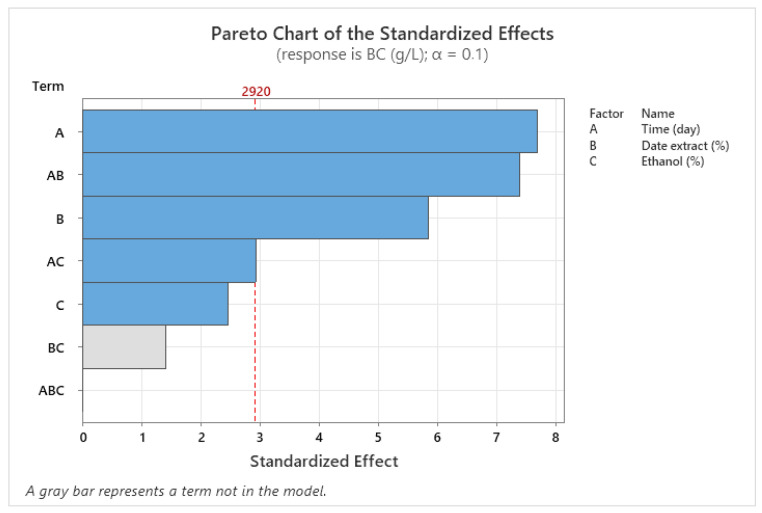
The Pareto chart of the standardized effects.

FFD was adopted to investigate the single and combined effects of three key factors -incubation time, date extract concentration and ethanol concentration—on BNC production yield. Analysis of variance was performed to assess the reliability of the proposed model in predicting the response value. As shown in Table 5, the model is statistically significant (*p*-value < 0.05), showing its capacity to reliably estimate the response value. The coefficient of determination (R^2^) reveals that 98.78% of the total variation can be explained by the model. Both the first-order factors (incubation time, date extract concentration) and interaction factor (incubation time and date extract concentration) significantly affected the BNC yield (*p*-value < 0.05). The Pareto chart confirmed these results further, where the factors crossing the reference line were considered the most significant (Figure 6). Therefore, the proposed model was represented by the following equation:

BNC (g/L) = 4.05 − 0.195 X_1_ − 1.400 X_2_ + 1.930 X_3_ + 0.2535 X_1_ × X_2_ − 0.2010 X_1_ × X_3_

**Table 5:** ANOVA of a full factorial design for BNC production by the strain IS22.

Source	DF	Adj SS	Adj MS	F-Value	*p*-Value
Model	5	9.5568	1.91135	32.50	0.030
Linear	3	5.8386	1.94621	33.09	0.029
X_1_	1	3.4716	3.47161	59.03	0.017
X_2_	1	2.0100	2.01001	34.18	0.028
X_3_	1	0.3570	0.35701	6.07	0.133
2-Way Interactions	2	3.7181	1.85906	31.61	0.031
X_1_ × X_2_	1	3.2131	3.21311	54.63	0.018
X_1_ × X_3_	1	0.5050	0.50501	8.59	0.099
Error	2	0.1176	0.05881		
Total	7	9.6744			

S = 0.242513, R^2^ = 98.78%, R^2^ (adj) = 95.74%. X_1_: Time (day), X_2_: Date extract (%), X_3_: Ethanol (%).

**Figure 7:**
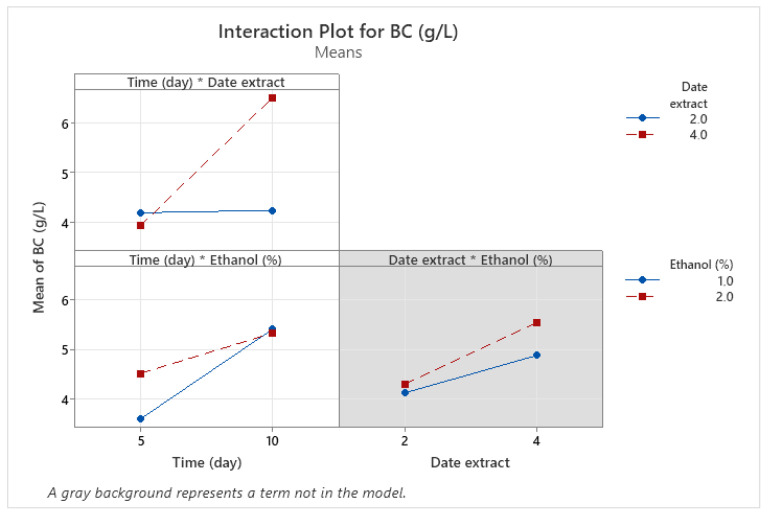
Interaction plot of time, date extract, and ethanol on BNC production yield. “* indicates the interaction between factors”.

For further insights about the influence of each variable and their mutual interaction on the target response, an interaction plot was constructed and is displayed in Figure 7.

When the response value varies from the low to the high levels of one factor based on the level of the second factor, this indicates the existence of an interaction between those factors. A stronger deviation of the lines indicates a stronger interaction. As shown in Figure 7, the BNC yield increased from 4 g/L to 6.5 g/L, as the incubation time increased from 5 to 10 days at a date extract concentration of 4%. The highest response value was obtained at a maximum date extract concentration. Meanwhile, the BNC yield remained stable from 5 to 10 days at 2% date extract. These findings confirm that a higher concentration of date extract promotes the biosynthesis of BNC. Similarly, Gendi et al. showed that the use of 68% of enzymatically hydrolyzed prickly pear peels (PPPE) as the sole source of nutrients yielded 6.01 g/L of BNC after 11 days of incubation at 20 °C, demonstrating the significant effect of carbon source concentration on BNC productivity [53]. In addition, the carbon source type affects the BNC synthesis. In HS medium, most of the glucose is typically converted into gluconic acid, leading to a pH drop, which significantly inhibits the BNC production. For this reason, date waste extract was used in the present study as an alternative carbon source, owing to its richness in sugars such as fructose and sucrose. These sugars generate different concentrations of gluconic acids, resulting in varying pH values. For instance, in a study carried out by Heydorn et al., the use of molasses as a sucrose-containing carbon source enhanced the BNC production by 1.2-fold compared to the standard media (HS) [54]. Similarly, several studies have confirmed that the consumption of carbon sources generates gluconic acid, which at higher concentrations may hinder the BNC production. Therefore, the use of some additives such as ethanol was demonstrated to solve the problem. For this reason, in this study, we tested the addition of ethanol at two levels to evaluate its impact on BNC yield. The interaction between time and ethanol revealed that the response increased from 3.85 g/L to 5.3 g/L, as the incubation time increased from 5 to 10 days, at 1% ethanol. At the same time, a similar pattern was observed with an increase from 4.5 g/L to 5.3 g/L at 2% ethanol concentration. In this case, ethanol concentration did not significantly affect the BNC yield. It means that both levels of ethanol resulted in the same BNC value after 10 days of incubation. These results were further confirmed by the ANOVA table, where the *p*-value was greater than 0.05.

Furthermore, the interaction between date extract and ethanol enhanced the BNC yield from 4 to 5 g/L, as the date extract concentration increased from 2 to 4% in the presence of 1% ethanol. Moreover, the BNC yield increased from 2.2 to 5.5 g/L using 2% ethanol. According to these results, we can conclude that 2% ethanol improves the BNC yield than 1% but not significantly (ethanol effect > 0.05). Our findings are consistent with those provided by Jacek et al., who observed a threefold increase in BNC yield after supplementing the culture media with 2% ethanol. This was explained by the fact that ethanol serves as an extra source of ATP and reduces spontaneous mutations in BNC-producing bacteria [55]. Other studies have also confirmed that the presence of ethanol in the culture media accelerates the conversion of glucose into bacterial nanocellulose via cellulose synthase, resulting in higher BNC yields [56,57]. Heydorn et al. demonstrated that the supplementation of HS media with 0.5% ethanol increased the BNC production from 1.7 g/L to around 4 g/L after 168 h [54].

**Figure 8:**
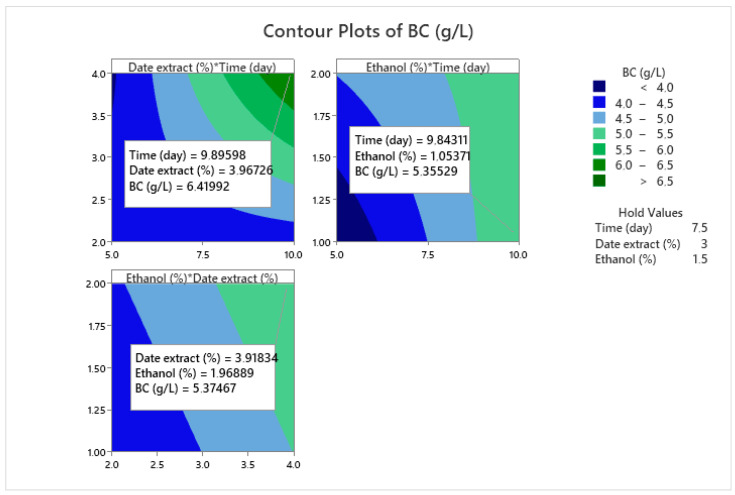
Contour plot of BNC production by the strain IS22. “* indicates the interaction between factors”.

**Figure 9:**
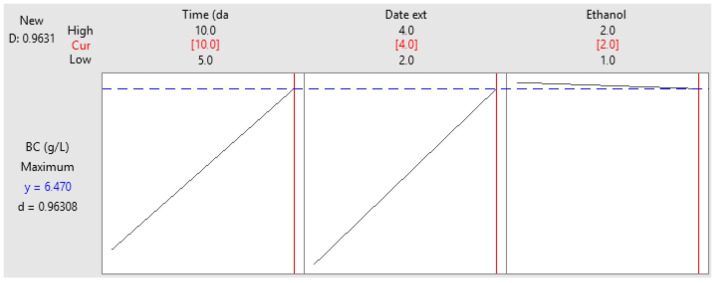
Optimum conditions for BNC production.

To identify the optimal conditions for maximum BNC yield, a contour plot was constructed. As shown in Figure 8, the highest BNC production was achieved using 3.5–4% of date extract, 1–2% ethanol and an incubation time of at least 9 days. The specific optimum conditions are summarized in Figure 9 as follows: 4% date extract, 2% ethanol and 10 days of incubation for the strain *Komagataeibacter* sp. IS22.

To validate the proposed model, the production process was performed in triplicate under the optimum conditions and compared to the predicted value. The BNC production yielded 6.25 g/L, which aligns well with the predicted value (6.48 g/L). These results were obtained with a desirability of 96.30% (Figure 9). In comparison to the standard media (HS), the production yield of BNC was improved by about threefold, after optimizing the culture conditions, using date extract as an alternative carbon source (from 1.8 to 6.59 g/L) and ethanol as a booster compared to the HS media. Thus, date extract prepared from date waste serves as a sustainable source to produce high amounts of BNC, reducing the production costs and supporting the concept of a circular economy. In the same context, various studies have investigated the use of different waste streams for BNC production. Among them, Raiszadeh-Jahromi et al. examined the effect of date syrup and cheese whey on BNC production yield. Results showed an optimum BNC yield of 1.88 g/L on the 10th day of cultivation using a 50:50 ratio of date syrup and cheese whey [58]. El-Gendi et al. also reported the production of 2.94 g/L of BNC using enzymatically hydrolyzed prickly pear peels as the sole production medium. After optimizing the production through a central composite design, it reaches 6.01 g/L after 11 days of incubation [53]. In addition, Hasanin et al. obtained a BNC yield of 1.262 ± 0.07 g/L using a new isolated *Achromobacter* sp. grown on mango peel waste hydrolysate as a low-cost culture medium [59]. Another study carried out by Rani and Appaiah showed a BNC production of 7.40 g/L using a new isolated, *Gluconacetobacter hansenii* UAC09, under static conditions with 4% glucose, 8% corn steep liquor (CSL) [60]. Overall, this study emphasizes the ability of a new isolated strain named *Komagataeibacter* sp. IS22 to metabolize different carbon sources and provide a high BNC yield.

After optimizing BNC production using waste extract and confirming the enhanced production yield, the next step was to explore the practical applications of the produced BNC. The final phase of this study focused on evaluating the potential of BNC as a carrier for probiotic microorganisms and assessing its functional performance, particularly in terms of viability and antimicrobial activity.

### 3.5. Application of BNC as Probiotic Carrier

#### 3.5.1. Viability of Immobilized Probiotic Cells

Bacterial nanocellulose is a versatile, biocompatible biomaterial with a porous nanofibrillar structure, ideal for immobilizing and encapsulating cells, enzymes, and probiotics while preserving their activity. The integration of probiotics into BNC is highly related to the type of cellulose, the production process, and the immobilization approach [61].

In this study, the BNC produced by *Komagataeibacter* sp. IS22 was evaluated for the immobilization of two probiotic strains, including *Bacillus subtilis* and *Lactobacillus plantarum,* through a co-culture approach.

Probiotics are live, non-pathogenic microorganisms that, when consumed in sufficient quantities, offer health benefits to the host by helping to restore the natural balance of the microbiota or by producing substances that inhibit harmful microbes [62]. Several studies have shown the positive effect of probiotics in the prevention or treatment of various diseases, such as acute viral gastroenteritis [63], inflammatory bowel diseases [64], pediatric post-antibiotic-associated diarrhea [65], and post-surgical pouchitis [66].

Results indicated that the numbers of free and immobilized cells within BNC pellicles were 8.18 log and 8.13 log CFU/mL for *B. subtilis*, and 7.83 log and 7.24 log CFU/mL for *L. plantarum,* respectively. These strains were able to adhere and survive within the fibrous nanostructure of bacterial nanocellulose without the need for chemical bonds. The cellulose ribbons were overlapped, interwoven, and arranged in parallel but disorganized, creating spaces that could trap bacterial cells both inside and on the surface, thereby improving their survival. It was also shown that the fibrous structure of BNC acts as a cryoprotectant, supporting the growth and the viability of probiotic cells [67]. Phromthep and Leenanon have investigated the survival of *Lactobacillus plantarum* immobilized in BC cubes prepared from mature coconut water. After 28 days of storage, the cell count revealed the survival of 6.41 log CFU/mL compared to 0.76 log CFU/mL for free cells [68]. Similarly, Jayani et al. revealed a survival rate of 71.1% for *L. acidophilus* 016 immobilized into BC nanofibers [69]. A further study showed the survival of recombinant *E. coli* cells within BC–silk fibroin composites after UV exposure, due to the ability of BC to absorb UV light and the biofilm-like environment provided by the scaffold [70].

#### 3.5.2. Antibacterial Activity of BNC-Probiotic Composites

**Figure 10:**
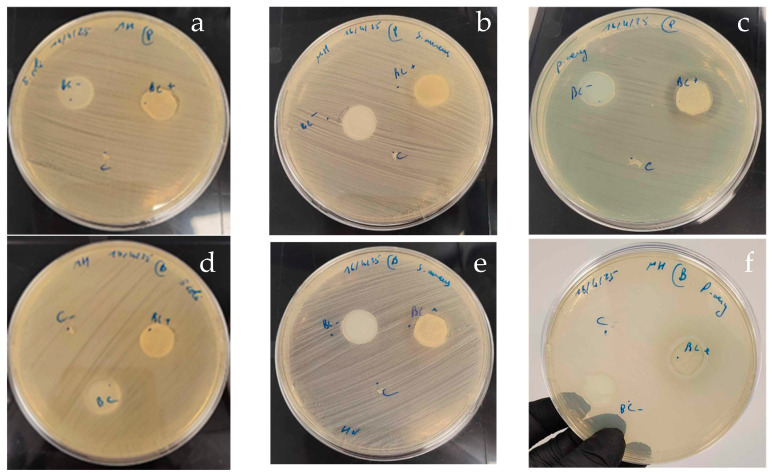
Inhibitory activity of probiotic-BNC composites. *Lactobacillus plantarum*, *Bacillus subtilis*, respectively, against *E coli* (**a**,**d**), *Staphylococcus aureus* (**b**,**e**), and *P. aeruginosa* (**c**,**f**). (BC+) BNC—probiotic composite; (BC-) negative control; (C) positive control (free probiotic).

The antibacterial activity of BNC–probiotic composites was evaluated using the agar diffusion assay on MHA against three pathogenic strains: *E. coli*, *S. aureus*, and *P. aeruginosa*. The results were compared to those obtained with non-encapsulated probiotic cells and neat (unmodified) bacterial nanocellulose. The agar diffusion experiments showed that both composites BNC-BS and BNC-LP exhibit an inhibitory activity against *P. aeruginosa* (Figure 10). Otherwise, BNC alone did not exhibit any inhibitory activity against all the tested pathogenic strains. Free probiotics strains also showed no inhibitory activity against the studied pathogenic strains. These findings may be explained by the fact that the cellulose nanofibrils provide an optimal environment for microbial adhesion and biofilm formation, which improves the probiotic colonization, stimulates their growth, and increases their metabolic activity. Gutiérrez-Fernández et al. have demonstrated that BC-encapsulated *Lactobacillus plantarum* effectively inhibits carbapenem-resistant *Klebsiella pneumoniae* and *Enterobacter cloacae*, while free-probiotic strains showed no inhibition [71]. Similarly, Sabio et al. have reported that probiotic cellulose significantly decreased the survival of *Staphylococcus aureus* and *Pseudomonas aeruginosa*, the primary pathogens responsible for severe skin infections and chronic wounds [26]. Another study in line with our findings revealed high antagonistic activity of *Bacillus subtilis* P-2 cells embedded within BNC towards *Staphylococcus aureus*, *Staphylococcus epidermidis*, *Escherichia coli*, and *Pseudomonas aeruginosa* [72].

Furthermore, research highlights the use of BNC—probiotic composites as innovative delivery systems, effectively maintaining probiotic viability and functional activity through food processing and storage. For instance, the use of BNC-probiotic composites in the dairy industry and fermented foods has been shown to enhance probiotics viability over storage and improve the sensorial and physicochemical properties of the final product. Lappa et al. reported that sour milk produced using BNC-probiotic composite presented higher organoleptic scores, compared to the free cells case, and enhanced probiotic viability during post-fermentation storage (4 °C, 28 days) [73]. In a similar study, Phromthep and Leenanon investigated the survival of immobilized *L. plantarum* within BNC cubes in mamao juice. Results showed that BNC maintained high probiotic counts of 6.32 log CFU/mL versus 0.76 log CFU/mL for the free cells after 28 days of storage, but also improved the overall organoleptic qualities of the beverage [63]. Moreover, probiotic encapsulation within BNC material could serve as an active food packaging material. For example, Moghanjougi et al. have successfully developed BNC composites containing free or microencapsulated probiotics (*L. acidophilus* or *Bifidobacterium animalis*) that inhibited the growth of *Aspergillus niger* in cheese [74].

In summary, the synergetic interaction between inert BNC fibrils and living probiotics improves their overall performance, which paves the way to their use in several applications, such as functional food applications, antimicrobial dressings, and drug delivery systems. However, further research remains essential to investigate effective encapsulation methods using bacterial nanocellulose and to use novel probiotic strains to expand the application of cellulose-based probiotic films.

The present study demonstrates, for the first time, the successful conversion of date waste—a readily available, low-cost agro-industrial by-product—into high-yield BNC using a newly isolated *Komagataeibacter* sp. IS22. While earlier studies have explored fruit-based substrates, the valorization of date waste remains underreported, especially in the context of simultaneous BNC production and probiotic delivery platforms development. This dual-purpose utilization underscores the sustainability and economic feasibility of the approach.

Furthermore, the selection of BNC as a probiotic carrier is supported by its biocompatibility, mechanical properties, and high surface area. However, the efficiency of probiotic delivery also depends heavily on the polymeric matrix properties. As discussed by Ntsefong et al.(2023) [75], optimal polymers for probiotic encapsulation should ensure protection against gastric acidity, controlled release, and viability preservation. Polymers such as alginate, chitosan, and pectin have been shown to offer these properties, either alone or in composite formulations. Integrating such polymers with BNC could enhance its functionality in gastrointestinal delivery, warranting future investigation.

This work thus not only presents a novel carbon source but also opens avenues for composite systems, combining BNC with other biopolymers for tailored probiotic release profiles.

## 4. Conclusions

In this study, we addressed two key limitations in the industrial production of BNC: the high production cost and the low yield. Specifically, we aimed to isolate high-producing BNC bacterial strains and to assess the valorization of date industry by-products as a renewable, low-cost carbon source.

Our results demonstrated that *Komagataeibacter* sp. IS22 is a particularly efficient BNC producer, reaching 1.8 g/L under standard conditions. Characterization of BNC confirmed the production of high-purity Iα cellulose with consistent nanofibrillar morphology across all strains. Importantly, the use of low-grade date waste extract as fermentation medium significantly enhanced BNC yield—up to 6.59 g/L after optimization via full factorial design—while aligning with circular economy strategies and reducing environmental impact.

Furthermore, the immobilization of probiotics within the BNC network enhanced their antibacterial activity against *Pseudomonas aeruginosa*. Additional research will address the control of the release rate of the embedded probiotics and investigation of the interactions with living cells, broadening their application in the food sector as well as biomedicine and other fields.

## Figures and Tables

**Table 1:** FFD experimental matrix of dry BNC weight (g/L).

Runs	X_1_	X_2_	X_3_	Experimental	Predicted
1	5 (−1)	2 (−1)	2 (+1)	4.53	4.66
2	10 (+1)	4 (+1)	2 (+1)	6.59	6.48
3	10 (+1)	2 (−1)	2 (+1)	4.08	4.21
4	5 (−1)	2 (−1)	1 (−1)	3.85	3.74
5	10 (+1)	2 (−1)	1 (−1)	4.4	4.29
6	5 (−1)	4 (+1)	1 (−1)	3.34	3.47
7	10 (+1)	4 (+1)	1 (−1)	6.43	6.56
8	5 (−1)	4 (+1)	2 (+1)	4.51	4.40

X_1_: Time (day), X_2_: Date extract (%), X_3_: Ethanol (%).

**Table 3:** Infrared crystallinity ratio and hydrogen bond intensity of the BNC pellicles.

Samples	TCI	LOI	HBI
	H1372/H2900	H1429/H897	A3400/A1320
IS20	0.999799	0.994601	0.996281
IS21	1.000803	0.9976	0.999198
IS22	0.996679	0.991493	0.992936

**Table 4:** Screening of different media compositions for BNC production.

Culture Media	BNC Yield (g/L)
HS	1.8 ± 0.25
HS + AE	2.81 ± 0.11
HS + KE	2.6 ± 0.08
AE	0.48 ± 0.01
KE	0.55 ± 0.01

(*p*-value < 0.001).

## Data Availability

The original contributions presented in this study are included in the article. Further inquiries can be directed to the corresponding authors.
